# Contraction Mechanisms in Composite Active Actin Networks

**DOI:** 10.1371/journal.pone.0039869

**Published:** 2012-07-02

**Authors:** Simone Köhler, Andreas R. Bausch

**Affiliations:** Lehrstuhl für Biophysik E27, Technische Universität München, Garching, Germany; University of Bristol, United Kingdom

## Abstract

Simplified *in vitro* systems are ideally suited for studying the principle mechanisms of the contraction of cytoskeletal actin systems. To shed light on the dependence of the contraction mechanism on the nature of the crosslinking proteins, we study reconstituted *in vitro* active actin networks on different length scales ranging from the molecular organization to the macroscopic contraction. Distinct contraction mechanisms are observed in polar and apolar crosslinked active gels whereas composite active gels crosslinked in a polar and apolar fashion at the same time exhibit both mechanisms simultaneously. In polar active actin/fascin networks initially bundles are formed which are then rearranged. In contrast, apolar cortexillin-I crosslinked active gels are bundled only after reorganization of actin filaments by myosin-II motor filaments.

## Introduction

In reconstituted *in vitro* actin networks, the simultaneous presence of myosin-II filaments and actin crosslinking proteins leads to a macroscopic contraction at high filament density [1], [2]. To lay the basis for understanding the physical principles of the macroscopic contraction, we investigate such reconstituted active actin systems with polar and apolar crosslinking proteins, where polar and apolar crosslinking proteins are distinguished by their organization of actin filaments in bundles: bundles built from polar crosslinking proteins such as fascin consist of parallel, unipolar actin filaments while the apolar crosslinking protein cortexillin organizes actin filaments in apolar bundles and crosslinked networks.

*In vivo*, contractile elements, which are essential for various cellular tasks as e.g. cytokinesis [3] or tissue morphogenesis [4]–[6], are built from actin filaments, myosin-II filaments and crosslinking proteins [7]. Only apolar crosslinking molecules are found in these cellular contractile elements [8]–[10]. Polar bundling proteins on the other hand side are employed in stable, non-contractile cellular structures [11]. Yet, it has been demonstrated that *in vitro* both kinds, polar and apolar crosslinking proteins suffice to induce a macroscopic contraction [2], [12].

Recently, the occurence of microscopic dynamics and the existence of a highly dynamic steady state could be demonstrated at low density of actin filaments in active *in vitro* actin networks which are crosslinked by the polar bundling protein fascin [2], [13]. It remains to be investigated how the nature of the crosslinking protein affects the mechanism of the emerging structures and dynamics and how the microscopic dynamics scale up to the macroscopic contraction mechanism.

To gain insight in the effect of the difference in crosslinking protein we investigate the contraction behavior of polar fascin [14] and apolar cortexillin-I [15] crosslinked active actin networks on different length scales ranging from the molecular organization to the macroscopic contraction.

We show that the macroscopic contraction of reconstituted active crosslinked networks depends on the microscopic structures of the contractile elements which in turn depend on the nature of the crosslinking molecule. Fascin induces a rapid bundling of actin filaments. These polar bundles can be rearranged subsequently by myosin-II filaments. By contrast, initial bundle formation in active cortexillin-I networks is effectively impaired by the presence of myosin-II and only much smaller clusters emerge. Thus the microscopic active cortexillin-I clusters are formed by individual filaments which are then bundled by cortexillin-I inside the clusters. As a macroscopic consequence cortexillin-I crosslinking results in a much more contracted active network than active fascin systems. More complex networks with a combination of both crosslinking molecules combine the typical structural and dynamic properties of the individual subsystems, yet no phase separation is observable.

## Results

**Figure 1:**
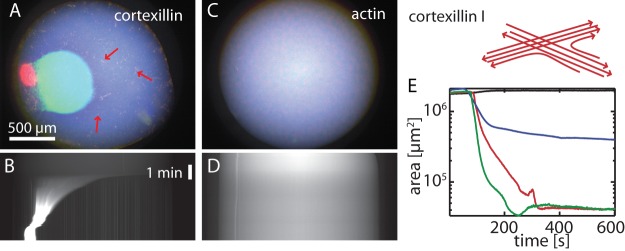
Macroscopic contraction of active actin/cortexillin-I networks. Droplets of 1.5 L of 10 M actin, 0.1 M myosin-II in presence (**A, B**) or absence (**C, D**) of 1 M cortexillin-I are shown as color overlay imediately (blue), 2 min (green) or 20 min (red) after initiation of polymerization (**A, C**). Macroscopic contraction is observed for active gels in presence of cortexillin-I while active actomyosin does not contract. Red arrows point to small clusters of secondary contractions. The time traces of the contractions are shown in the kymographs (**B, D**). The quantitative analysis of the contraction (**E**) shows, that active gels in presence of the apolar crosslinking protein cortexillin-I contract to smaller areas than in presence of fascin alone albeit at similar time scales (black: no crosslinker; red: 1 M cortexillin; blue: 1 M fascin; green: 1 M cortexillin-I and fascin, each).

To investigate the contraction of cortexillin-I crosslinked active gels, 1.5 L droplets of the active gel solution embedded in oil are observed (Fig. 1). Cortexillin-I has been shown to crosslink actin filaments in random orientation to form networks and antiparallel or, less frequently, parallel bundles [15]. Therefore we refer to cortexillin-I as an apolar bundling protein.

Actin networks (10 M) crosslinked by cortexillin-I (1 M) show a rapid macroscopic contraction immediately after polymerization when myosin-II filaments (0.1 M myosin) are present (Fig. 1A–B and video S1). The contraction reduces the observable projection area of the total network down to 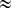
3%. In absence of any crosslinking protein, the actomyosin gel remains homogeneous and no macroscopic contraction is observed (Fig. 1C–D and video S2). This is in accordance with the fact that crosslinking proteins are essential for the macroscopic contraction [1]. Similarily, actomyosin in presence of low cortexillin-I concentrations (0.1 M) does not contract (Fig. S1). Strongly increasing the cortexillin-I concentration to 10 M reduces the contractility of the active gels: the contraction starts at later times and does not proceed as far as observed for intermediate cortexillin-I concentrations (Fig. S1). Likewise, macroscopic contractility is suppressed in presence of high 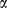-actinin concentrations [1].

**Figure 2:**
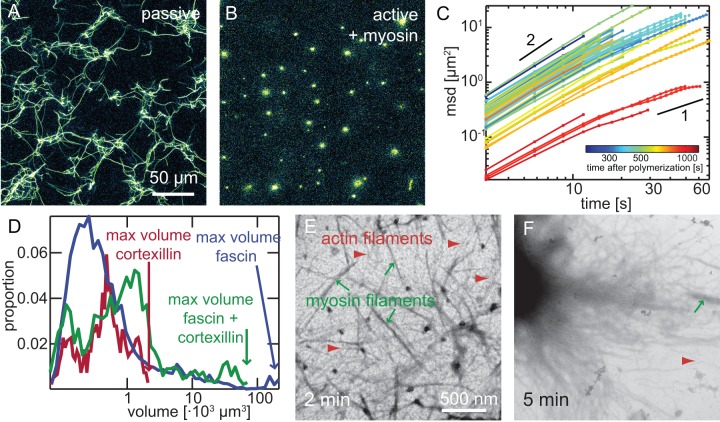
Microscopic structure of active actin/cortexillin-I networks. Confocal micrographs of passive (**A**) or active (**B**; 0.1 M myosin) actin networks (3 M actin) crosslinked by 1 M cortexillin. In the passive state, an inhomogeneous bundle network is formed (**A**). In presence of myosin-II filaments, small clusters emerge (**B**). Clusters are traced over time in 3D and the mean square displacements of these traces are shown in **C**. Colors from blue to red denote the starting time of the trajectories with respect to initiation of polymerization. All clusters initially show superdiffusive behavior. The apparent diffusion constant as given by the intercept decreases for clusters at late times. The histograms of cluster volumes (**D**) obtained from confocal micrographs (shown is a histogram over all times normalized to the number of clusters *N* found in a 456x456x50 m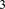 confocal volume) demonstrate, that the maximal cluster size (arrows) increases from apolar cortexillin-I active gels (red, *N* = 1366) over composites of fascin and cortexillin-I (green; 0.5 M each, *N* = 1318) to unipolar bundled actin/fascin/myosin networks (blue, 1 M fascin, *N* = 5260) by two orders of magnitudes. Electron micrographs reveal, that in active actin/cortexillin-I networks myosin-II filaments first reorganize actin filaments (**E**, 2 min after initiation of polymerization). Actin filaments (red arrowheads) can readily be identified by their long, thin helical structure, while myosin-II filaments (green arrows) are significantly thicker and concomitantly darker. Actin/cortexillin-I bundles start to emerge in reorganized regions. The reorganization results in the formation of clusters (**F**, 5 min after initiation of polymerization), which are further condensed by cortexillin-I mediated bundling.

To further investigate the molecular mechanism of the contraction in presence of cortexillin-I, we analyze the microscopic structure of both the active and a passive actin/cortexillin-I system at a lower actin concentration (3 M) using confocal microscopy (Fig. 2A–B). In the passive state without myosin-II motor filaments, cortexillin-I bundles actin in an apolar fashion to form an inhomogeneous network of patches with dense bundles (Fig. 2A).

The addition of myosin-II filaments results in the formation of active gels with marked reorganization dynamics: instead of a bundle network, only small clusters are generated (Fig. 2B). Initially, very small clusters are formed. These small clusters are actively rearranged in a stop and go mode. Multiple fusion events give rise to the formation of larger clusters. These larger clusters show continuing stop and go motion for at least 40 min after initiation of polymerization (Fig. S2A and video S3). To quantify the dynamics in the active cortexillin-I network, we analyze the mean square displacement of the threedimensional trajectories of individual clusters (Fig. 2C). All trajectories show superdiffusive behavior. This superdiffusivity is characterized by a power law exponent of the mean square displacement larger than 1 but smaller than 2 [16], [17]. For some traces, a loss of superdiffusivity on long time scales is observed which is more frequent at late times. This is also found in active actin/fascin networks where it has been attributed to local rearrangements in otherwise immobile clusters [2]. Due to their small size, local rearrangements cannot be resolved in actin/cortexillin/myosin clusters, but the loss of superdiffusivity at long time scales might result from an increase in stalling events in the steady state at late times. The apparent diffusion constant as observed in the intercept of mean square displacements, decreases with the age of the network (Fig. 2C, blue to red) but not with the cluster size (Fig. S3). This might be attributed to the background network.

The analysis of the distribution of cluster volumes reveals two peaks (Fig. 2D, red line): The initial cluster formation process by fusion of many small clusters results in the occurrence of a peak at small volumes of about 0.1 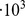 m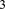 in the histogram of cluster volumes. Fusion events driven by the activity of myosin-II motor filaments result in larger clusters (video S3). The maximal cluster volume observed amounts to 2 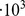 m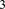 (red arrow).

Due to the optical resolution limit, the molecular structure and the initial formation mechanism of actin/cortexillin/myosin clusters cannot be resolved by confocal microscopy. To this end, we use time resolved transmission electron microscopy. The first step of the cluster formation in actin/cortexillin-I/myosin gels is the formation of actin filaments (Fig. 2E, red arrowheads) within a myosin-II network (green arrows, Fig. 2E, 2 min after initiation of polymerization and Fig. S4A). These filaments are immediately rearranged into clusters and then reorganized into bundles (blue arrows Fig. S4A). Myosin-II filaments can be included in such clusters (green arrow Fig. S4A, 5 min). Bundles are not observed prior to cluster formation but only within clusters. This indicates, that the minimal building blocks in this system are filaments. Individual clusters are connected via ‘arms’ consisting of several actin filaments (Fig. 2F, 5 min after initiation of polymerization). These ‘arms’ provide the basis for cluster-cluster fusion events as observed by confocal microscopy and might provide the tracks to ensure the stop and go motion of these clusters.

**Figure 3:**
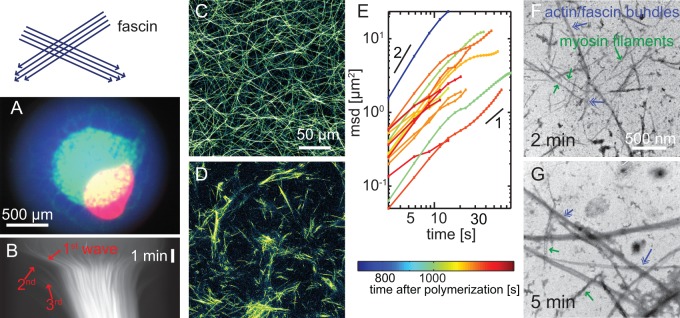
Active actin/fascin gels. Active gels crosslinked by fascin show a macroscopic contraction (**A**, color overlay of fluorescence micrographs imediately (blue), 2 min (green) or 20 min (red) after initiation of polymerization). This contraction occurs in multiple waves as can be seen in the kymograph (**B**, red arrows). Confocal micrographs of passive (**C**) actin/fascin networks (3 M actin, 1 M fascin) show long and homogeneously sized bundles. In presence of myosin-II filaments (0.1 M; **D**) large structures consisting of several bundles are formed. Mean square displacements of 3D-trajectories of individual actin structures (**E**) show superdiffusive behavior of the clusters. Colors denote the time after initiation of polymerization. Please note that mean square displacements can only be calculated for long time traces which are not recorded initially due to large run lengths which are not fully captured by the high magnifications used here. The dynamics in active actin/fascin networks have been described in great detail in 2D in [2], [13] and are in accordance with the results presented here. Electron micrographs of active networks in presence of fascin show, that actin/fascin bundles (blue arrows) are formed immediately after initiation of polymerization (**F**, 2 min), which are then reorganized into clusters by myosin-II filaments (green arrows, **G**, 5 min).

*In vivo*, actin structures crosslinked with unipolar bundling proteins are utilized to build stable and non-contractile structures [11]. On the other hand, a macroscopic contraction is observed when using the unipolar bundling protein fascin in active gels at high concentrations *in vitro* (Fig. 3A–B and video S4). This contraction occurs on time scales comparable to active gels crosslinked by cortexillin-I (Fig. 1E and Fig. S1). However, the extent of the contraction depends critically on the nature of the bundling protein. Active gels crosslinked by the unipolar bundling protein fascin contract to 15% of their initial area (Fig. 1E, blue line and Fig. S1), thus much less than the contraction observed for the apolar bundling protein cortexillin-I. The progress and the amount of the contraction do not correlate with the fascin concentrations (Fig. S1) which is in marked contrast to the concentration dependence of the macroscopic contraction in active apolar cortexillin-I (this study) or 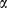-actinin [1] actin networks.

Remarkably, we find multiple contraction waves in presence of sufficiently high fascin concentrations (Fig. 3B, red arrows and video S4). This is not observed in presence of cortexillin-I (Fig. 1B). These contraction waves are characterized by successive contraction events with a decrease in actin content as can be seen in a decay in fluorescence intensity. On a microscopic scale, fascin organizes actin filaments into a homogenous network of long, straight and unipolar bundles with uniform thickness (Fig. 3C) [18]. The addition of myosin-II filaments results in the formation of active gels with marked reorganization dynamics (Fig. 3D and video S5). These dynamics are characterized by superdiffusive behavior of the actin bundle structures (Fig. 3E and Fig. S2B) [2]. Therefore, the basis for the observed macroscopic contraction of active actin/fascin gels might be the random orientation of the individual bundles which cannot be disintegrated due to their unipolar filament alignment. The dynamic rearrangement of these active actin/fascin network results in the formation of large clusters from individual bundles (Fig. 3D and video S5). Their maximal volume amounts to at least 200 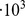 m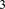 (Fig. 2D). Please note that the field of view is not large enough to measure the maximal size of actin/fascin/myosin clusters with the setup used here, yet they are significantly larger than clusters formed in active actin/cortexillin-I systems. Different mechanisms are conceivable to explain this difference in cluster volume: (*i*) The clusters are less densely packed in presence of fascin than in presence of cortexillin. This is supported by the fact that active actin/fascin gels contract to a lesser extent than apolar crosslinked active networks (Fig. 1E and Fig. S1). (*ii*) Furthermore, the observed larger cluster volumes also arise from a larger interaction length of the clusters for fusion events. While cluster-cluster fusion in active cortexillin-I networks is mediated by actin filaments, the interaction range of active actin/fascin clusters is larger by unipolar actin/fascin bundles which cannot be disintegrated by myosin-II filaments.

Electron microscopy is used to test both microscopic mechanisms. In presence of fascin, first actin/fascin bundles appear (2 min after initiation of polymerization, Fig. 3F and Fig. S4B, blue arrows). These bundles are rearranged by myosin-II filaments (green arrows) resulting in the formation of larger structures (Fig. 3G). This structure formation with actin/fascin bundles as minimal building blocks is also observed in fluorescence microscopy. The actin/fascin bundles remain intact as their polar structure prevents any disintegration. In contrast to the cortexillin-I system, no single filaments are observed in presence of fascin. This indicates that active actin/fascin networks indeed use actin/fascin bundles as minimal building block. Thus, the larger cluster volumes in fascin networks, compared to apolar active networks results from less effective packing of the stiff polar bundles compared to the packing of apolar filaments and the concomitant larger interaction length of the bundles.

While the contraction behavior in actin/fascin and actin/cortexillin-I active gels occurs on similar time scales, they differ in their minimal building block: In unipolar actin/fascin active gels, only bundles are rearranged by myosin-II whereas single actin filaments are the minimal building block in apolar actin/cortexillin-I active gels. This raises the question how the contraction occurs in composite active networks with both crosslinking molecules. For these composite active gels several mechanisms are conceivable: Either one or the other crosslinking protein dominates, or both minimal building blocks occur simultaneously or a phase separation occurs.

**Figure 4:**
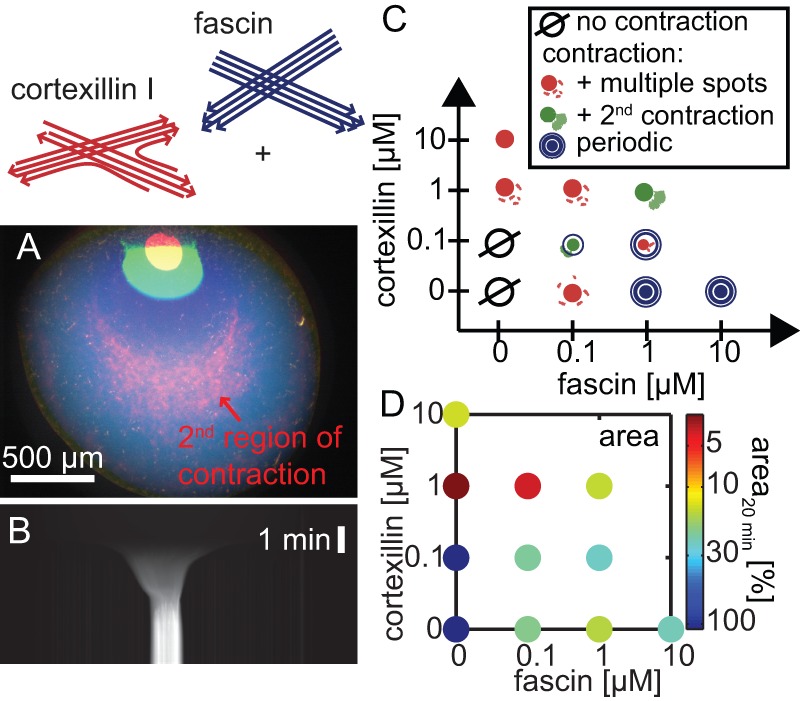
Composite active gels crosslinked by cortexillin-I and fascin. A drastic macroscopic contraction (**A, B**) is observed in active gels crosslinked by both, cortexillin-I and fascin (10 M actin, 0.1 M myosin-II, 1 M cortexillin-I and fascin, each). Fluorescence micrographs of the contraction are shown as color overlay imediately (blue), 2 min (green) or 20 min (red) after initiation of polymerization (**A**) and as kymograph (**B**). The red arrow indicates the second region of contraction. The phase diagram of contraction (**C**) illustrates, that active gels in presence of sufficiently high crosslinker concentrations contract macroscopically. Multiple contraction waves are observed when the polar bundling protein fascin dominates. The presence of cortexillin-I lowers the connectivity in the active gel and thus inhibits the ocurrence of multiple contraction waves. Instead secondary regions of contractions are observed. The final area of the contracted state depends on the crosslinker concentrations (**D**). The size of the contracted actin containing region with respect to the initial droplet area at 20 min after initiation of polymerization are shown. Please note, that in presence of 1 M fascin and cortexillin, each, the area corresponds to both, the first and second regions of contraction.

Using fascin and cortexillin-I composites at high actin concentrations, cortexillin-I dominates the macroscopic contraction (10 M actin, 1 M fascin and cortexillin-I, each; Fig. 4A–B and video S6): the extent of the contraction is as large as for cortexillin-I alone (Fig. 1E). The contraction is slightly faster than for both crosslinkers alone. Multiple contraction waves as observed in presence of fascin alone do not occur. Instead, a second spatially separated contracted region in the already-contracted area is found (Fig. 4A, arrow and video S6).

The dependence of the macroscopic contraction behavior on the crosslinker concentration is complex (Fig. 4C): Decreasing the cortexillin-I concentration (0.1 M cortexillin, 1 M fascin) induces multiple contraction waves. Decreasing the fascin concentration (1 M cortexillin, 0.1 M fascin) on the other hand results in the occurence of multiple secondary regions of contractions as observed in pure active cortexillin-I networks. Similarily, the area of the contracted area 20 min after initiation of polymerization is less than 5% of the initial area in presence of 1 M cortexillin-I (Fig. 4D and Fig. S1). This is also true for 1 M cortexillin-I and fascin, each. While the primary contracted region is contracted to 2% of the initial area (Fig. S1), the emergence of a secondary contracted region results in an apperently larger area of contracted regions 20 min after initiation of polymerization (Fig. 4D). In the presence of low cortexillin-I concentrations (0.1 M), the decrease in area upon contraction is comparable to that in pure active fascin networks.

The velocity of the macroscopic contraction ranges from −1.0%/s to −5.7%/s (Fig. S1, blue lines). Maximal velocities are observed at intermediate crosslinking protein concentrations (Fig. S5). While contraction velocities in prescence of the polar crosslinking protein fascin alone are relatively slow (about −1%/s), addition of small concentrations of fascin to active actin/cortexillin-I networks significantly increases the contraction velocity of the composite systems.

**Figure 5:**
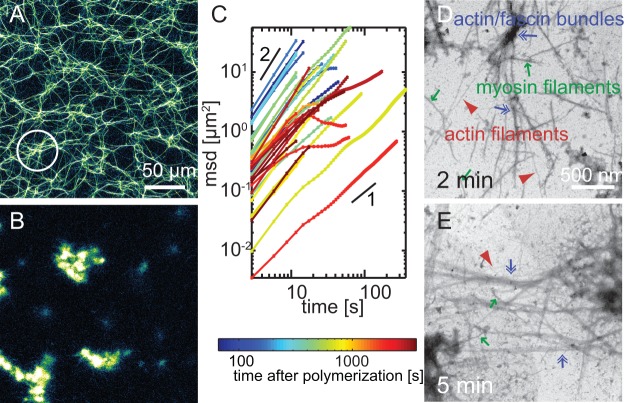
Microscopic structure of composite active gels. Confocal micrographs of passive (**A**) or active (**B**; 0.1 M myosin) actin networks (3 M actin) crosslinked by 0.5 M cortexillin-I and fascin are shown. The passive composite fascin/cortexillin-I network ressembles both pure fascin and cortexillin-I networks. Long, straight bundles are similar to those observed in actin/fascin networks, while clusters (white circle) resemble actin/cortexillin-I networks. Active gels crosslinked by cortexillin-I and fascin form clusters similar to those observed in actin/cortexillin/myosin networks albeit larger in size. The clusters exhibit superdiffusive dynamics at all times as shown in the mean square displacements in **C**. Electron micrographs of these active networks crosslinked by composites of fascin and cortexillin-I resemble both, pure fascin and pure cortexillin-I active networks: initially, bundles (blue arrows) as well as filaments (red arrowheads) are rearranged by myosin-II filaments (green arrows, **D**, 2 min) resulting in clusters of bundles supplemented by bundled filaments (**E**, 5 min).

The connectivity and the structure of the composite active gels is determined by confocal and electron microscopy at a lower actin concentration (3 M). In the passive composite network, neither fascin nor cortexillin-I dominates, but the resulting network resembles both (Fig. 5A): The bundles resemble pure actin/fascin networks but tend to be shorter and less uniform. Additionally, clusters similar to those observed in actin/cortexillin-I networks are formed (Fig. 5A, white circle). This is in accordance with composite passive filamin/fascin networks where both crosslinkers modify the network structure independently [19].

In active composite gels, actin structures are condensed to clusters (Fig. 5B and video S7). The structure of the clusters is reminiscent of an accumulation of clusters formed in active cortexillin-I networks. However, their volume is significantly larger than in presence of cortexillin-I alone; yet their maximal volume of 75 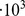 m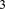 remains smaller than in presence of fascin alone (Fig. 2D). Similar to active actin/cortexillin-I or active actin/fascin networks, the clusters formed in active composites show stop and go dynamics (Fig. S2C).

On an even smaller length scale in the electron microscope, the nature of the minimal building block can be resolved: clusters are formed from filaments (Fig. 5D and Fig. S4C, 2 min, red arrowheads) as minimal building block comparable to the active cortexillin-I system. However, also individual bundles (blue arrows) contribute to cluster formation by myosin-II filaments (green arrows), as observed in the active fascin system. Cluster-cluster interaction is mediated by ‘arms’ partly consisting of bundles (Fig. 5E). This might result in a larger distance range for the interaction, as compared to the pure active cortexillin-I system. By that, more clusters can fuse resulting in the formation of larger clusters. This is consistent with the higher cluster size in the active composite network than in the pure active cortexillin-I gel, as observed with confocal microscopy (Fig. 2D). Thus, as observed in the passive composite network, the active gel crosslinked by fascin and cortexillin-I is built from both minimal building blocks in a composite manner.

## Discussion

The choice of crosslinking molecules determines the dynamics and structure of the active gel. Parallel bundling proteins, such as fascin, result in a highly dynamic steady state at small actin concentrations. The cluster sizes in this dynamic steady state show a distinct distribution resulting from an intricate balance between a crosslinker induced stabilization mechanism and the simultaneous destabilization processes by molecular motors. The emerging structures span sizes up to 100s of microns, due to the properties of the fascin bundles. In contrast, significantly smaller clusters are formed in active gels crosslinked by cortexillin-I in an apolar manner. These clusters only grow up to couple of microns and are only weakly connected with each other at the low actin concentrations studied here. The dynamics of the clusters is dominated by fusion events. Although the dynamics is clearly superdiffusive, the limited motion and statistics prohibits an in depth analysis of the trajectories.

**Figure 6:**
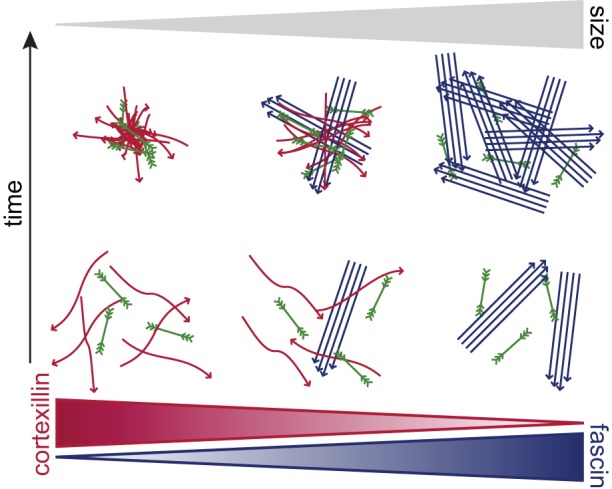
Contraction scenarios in composite active networks. A schematic overview of the contraction mechanisms in polar, apolar and composite active actin networks is shown.

At higher concentrations of actin, a macroscopic contraction is observable, which is most effective in the presence of the apolar crosslinking molecule. Thereby, the actin filaments are first contracted by the myosin-II filaments and subsequently stabilized into dense clusters by cortexillin-I (Fig. 6). These findings are consistent with the reformation of stress fibers after removal of blebbistatin in U2OS osteosarcoma cells [20]: 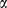-actinin presumably binds only after the reorganization of actin to thin bundles by myosin-II filaments. In order to produce contractile elements like stress fibers or contractile rings, stabilizing points are essential to be able to exert directional forces onto the filaments [21]. Importantly, an elasticity provided by either crosslinking molecules or other anchor points, such as adhesion sites on beads [22], is essential to enable the force exertion of motors to the network.

By contrast, the unipolar bundling protein fascin is opposing the contraction of individual filaments by the immediate stabilization into actin bundles. Subsequently myosin-II acts on these large structures, which sets the cluster sizes and limits the effectiveness of the macroscopic contraction (Fig. 6). The observed difference in the length scale of the contraction prompts to speculate, that this is a mechanism which might be employed *in vivo*: large structures of polar bundles might not be able to contract inside cells while the small structures built from filaments in apolar active gels are ideally suited to facilitate localized contraction events as found *in vivo* for example during cytokinesis [3]. Indeed, *in vivo* polar bundles are less prone to be used to produce contractile elements in cells, but are mostly found in stable structures like filopodia [11]. In cytokinetic *Dictyostelium discoideum* cells fimbrin, another unipolar bundling protein, is located predominantly globally at the non contracting pole regions, while cortexillin-I is found in the contracting equatorial region [23].

The length scales determining the connectivity in the active gels is set by the type of crosslinking protein: the minimal building blocks in polar active actin/fascin networks are actin/fascin bundles. Only in these systems, we observe multiple contraction waves in our minimal model system. Similar periodic contractions have been observed in mitotic cell extracts, where this phenomenon has been attributed to a gel growth model [24]: First, an actin gel is formed which spans the full volume. This network starts to contract after exceeding a critical connectivity. Actin monomers or filaments are not completely depleted from the already-contracted region but continue to grow resulting in the reformation of an actin gel. Once this second gel has reached the critical connectivity, a second wave of contraction emerges. The observed periodicity is the result of constant nucleation and elongation of remaining actin in the already-contracted region forming new contractile gels of decreasing density. While periodic contractions can proceed for up to 6 hours in cellular extracts [24], only few contracting waves are observed in our reconstituted system. This can easily be explained by the fact, that unlike cellular extracts, the minimal system lacks any factors accelerating the actin depolymerization and treadmilling.

Multiple contractions are not observed in active cortexillin-I networks (Fig. 1A–B). Yet, multiple small spots form in the already-contracted area of actin/cortexillin-I/myosin networks (Fig. 1A, arrows). These spots might be the result of local contraction similar to the formation of small clusters in the dilute regime because the connectivity in the already-contracted area of active cortexillin-I networks is not high enough to allow for multiple contraction waves. Consequently, the observed higher directional connectivity in active actin/fascin networks compared to active cortexillin-I networks should allow for a macroscopic contraction of polar bundled networks at lower actin concentrations than of apolar active gels. Indeed, active fascin networks still contract at an actin concentration of 7.5 M actin (video S8) but not active cortexillin-I gels (Fig. S6).

In composite active gels, both minimal building blocks coexist resulting in an intermediate contraction behavior: while the connectivity is not high enough for periodic contractions it nevertheless allows for a secondary region of contraction. Due to the combination of both types of minimal building blocks in composite active networks, their connectivity can be tuned by varying the concentrations of both crosslinking molecules giving rise to distinct intermediates in the contraction behavior (Fig. 4C): at low cortexillin-I concentrations (0.1 M) fascin dominates and periodic contractions occur albeit small clusters or a secondary contracted region are also observed. Increasing the cortexillin-I concentration and thereby decreasing the connectivity abolishes any multiple concentric contractions but results in secondary regions of contraction.

The spatial separation of the two regions of contraction can be attributed to the low connectivity between the areas: the newly formed actin filaments in the already-contracted region are disconnected from the major contracted network. Consequently, the newly polymerized active gel contracts to a second, less dense spot. By contrast, the minimal building block in pure active actin/fascin networks are parallel bundles resulting in long range interactions. In consequence, multiple contraction waves are observed due to the high connectivity. No waves of contraction are observed in presence of cortexillin-I alone, where the directional high connectivity is missing. Instead, many much smaller secondary regions of contractions can be identified in the active cortexillin-I gel (Fig. 1A, arrows). Thus, the emergence of a second contracted region in composite active gels with both, apolar and unipolar crosslinking proteins might stem from an intermediate connectivity between the clusters.

The simultaneous presence of two types of crosslinking molecules combines the typical structural and dynamic properties that are observed for each pure cross-linked system in the passive as well as in the active state (Fig. 6). This independent modification has already been observed for the mechanics of a passive composite network of filamin and fascin [19]. Although a phase separation of the structures would have been conceivable especially in the active state, only mixed states are observable. It remains to be explored if a temporal and local activation of constituents can already lead to stable and distinguishable phases [25], which would underline the importance of local and temporal separated activation of the actin binding proteins *in vivo*.

## Materials and Methods

### Protein purification

Myosin-II [26] and G-actin [27], [28] are extracted from rabbit skeletal muscle. Recombinant human fascin is purified from *E. coli* BL21-CodonPlus-RP and stored at −80°C in 2 mM Tris/HCl (pH 7.4), 150 mM KCl at 64 M [29]. Recombinant *Dictyostelium discoideum* cortexillin-I (gift from G. Gerisch, Max Planck Institute of Biochemistry, Germany) is purified from *E. coli* BL21-CodonPlus-RP using a C-terminal His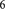-Tag and stored at −80°C in 20 mM Tris/HCl (pH 8.0), 100 mM NaCl, 4 mM CaCl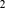 and 2 mM DTT [15].

### Fluorescence imaging

Active gels contain 10 M actin, 0.1 M myosin-II and indicated crosslinker concentrations in polymerization buffer (10 mM imidazole, 0.2 mM CaCl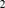, 3 mM MgCl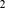, 10 mM DTT, 1 mM ATP). Actin filaments are labelled with 0.6 M Alexa Fluor 488-phalloidin (Invitrogen). To prevent any surface interaction, 2 mg/mL casein is added. The ATP concentration is kept constant by adding an ATP regeneration system (20 mM creatine phosphate and 0.1 mg/mL creatine phospho kinase (Sigma)). 1.5 L droplets are embedded in dodecane to eliminate any evaporation or drift in the samples and imaged on a Zeiss Axiovert 200 inverted microscope with a 10×(NA 0.2) long distance objective. Frames are captured at 1.19 s with a charge-coupled device camera (Orca ER, Hamamatsu) attached to the microscope via a 0.4×camera mount.

Confocal time lapse xyz-stacks are obtained by imaging 3 M actin labelled with 0.3M Alexa Fluor 488-phalloidin, 0.1 M myosin-II and indicated crosslinker concentrations in polymerization buffer with casein and an ATP regenerating system on a Leica TCS SP5 confocal microscope using a 63×(NA 1.4) oil immersion objective or 20×(NA 0.7) oil immersion objective to obtaine cluster size distributions. Samples are enclosed to hermetically sealed chambers to eliminate any drift in the network. We used the same magnification for all samples to ensure comparability between different samples. Indeed for fascin an even lower magnification would be needed to resolve all dynamics as addressed in 2D in [2], [13].

### Image processing

To quantify the dynamics of the macroscopic contraction, fluorescence images are corrected for inhomogeneous illumination by dividing by a 2-dimensional gaussian. To identify actin containing regions, a cutoff value is applied to these corrected images resulting in binary images. The cutoff value is obtained by determining the mean intensity values at positions of maximal intensity gradient corresponding to the borders of the actin containing regions. The area of these regions is calculated and compared with the maximal area of the droplet to obtain the percentage of contraction.

Cluster trajectories and cluster volume distributions are obtained from confocal xyz-stacks by identifying individual clusters using a cutoff value to generate binary stacks. Clusters are connected bright pixels larger than 34 m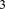 corresponding to a sphere with 4 m diameter. The clusters are traced over time using the IDL tracking algorithm [30] for the intensity weighted centroid cluster positions in three dimensions using Matlab R2008b (The MathWorks, Inc.). The mean square displacement is calculated for trajectories longer than 45 subsequent time points and the first 10% are shown. Cluster volume histograms are calculated for the maximal volumes of all clusters at all time points and normalized to the total number of clusters found.

### Electron microscopy

Samples containing 3 M actin, 0.1 M myosin-II and indicated concentrations of crosslinker in polymerization buffer are attached to grids (FCF400-Cu, EMS) at indicated times after initiation of polymerization for 1 min and negatively stained with uranylformiate.

## Supporting Information

Figure S1 **Dependence of the macroscopic contraction on the crosslinker concentration.** The decrease in area over time (red dots) are shown for 10 M actin, 0.1 M myosin and crosslinking molecules at concentrations as indicated by the green axis. The area of the contracted region is normalized to the maximal area in the non-contracted state. Blue lines denote linear fits to initial contraction velocities. Initial increases of the area are due to spreading of the droplets.
(TIF)

Figure S2 **Cluster trajectories in active actin networks.** Trajectories of clusters in active actin gels (3 M actin, 0.1 M myosin) are shown for networks crosslinked by 1 M cortexillin-I (**A**), 1 M fascin (**B**) and 0.5 M cortexillin-I and fascin, each (**C**). All trajectories exhibit stop and go motions.
(TIF)

Figure S3 **Dependence of the mean square displacement on the cluster volume.** Colors from blue to red denote the cluster volume. All clusters show superdiffusive behavior which does not correlate with volume.
(TIF)

Figure S4 **Time resolved electron micrographs of active actin networks.** Electron micrographs are shown at 2 min, 3 min, 5 min and 20 min after initiation of polymerization for 3 M actin, 0.1 M myosin and 1 M cortexillin-I (**A**) or fascin (**B**) or 0.5 M fascin and cortexillin, each (**C**), respectively. Red arrowheads point to actin filaments, blue arrows indicate actin bundles and green arrows show myosin-II filaments.
(TIF)

Figure S5 **Phase diagram of the macroscopic contraction velocity.** Initial velocities of contractions as shown in Fig. S1 are shown in dependence of crosslinker concentrations.
(TIF)

Figure S6 **Contraction scenarios in composite active networks.** A schematic overview of the contraction mechanisms in polar, apolar and composite active actin networks is shown.
(TIF)

Video S1 **Macroscopic contraction of active cortexillin-I networks.** A droplet (1.5 L of an active actin/cortexillin-I network (10 M actin, 1 M cortexillin-I, 0.1 M myosin-II) shows a rapid macroscopic contraction.
(MOV)

Video S2 **Actomyosin solution.** Droplets of actomyosin (10 M actin, 0.1 M myosin-II) are stable over time and do not show any contraction.
(MOV)

Video S3 **Cluster formation in active actin/cortexillin-I networks.** Average intensity *z*-projections of 50 m of 3 M actin, 1 M cortexillin-I and 0.1 M myosin-II are shown. Please note that the apparent disappearance of clusters is due to fusion with clusters outside the visualized stack.
(MOV)

Video S4 **Macroscopic contraction of active fascin networks.** A macroscopic contraction is observed in droplets of active actin/fascin networks (10 M actin, 1 M fascin, 0.1 M myosin-II). The high connectivity in these networks allows for multiple contraction waves.
(MOV)

Video S5 **Microscopic reorganization in active fascin networks.** Active actin/fascin networks at low actin concentrations (3 M actin, 1 M fascin, 0.1 M myosin-II) exhibit a marked reorganization dynamics (50 m *z*-projections are shown).
(MOV)

Video S6 **Macroscopic contraction in active composite networks.** Composite active networks crosslinked by cortexillin-I and fascin (10 M actin, 1 M fascin, 1 M cortexillin-I, 0.1 M myosin-II) contract to ≈3% of the initial area. The already-contracted region contracts to a secondary region.
(MOV)

Video S7 **Microcontraction in active composite networks.** At low actin concentrations, active composite actin networks (3 M actin, 0.5 M fascin, 0.5 M cortexillin-I, 0.1 M myosin-II) are rearranged into clusters, which are significantly larger than in presence of cortexillin-I alone (50 m *z*-projections are shown).
(MOV)

Video S8 **Macroscopic contraction of active fascin networks at low actin concentrations.** Active actin/fascin networks show a macroscopic contraction already at an actin concentration of 7.5 M (1 M fascin, 0.1 M myosin-II).
(MOV)
