# Supplementary figures and images for: Contraction Mechanisms in Composite Active Actin Networks
Source: PLoS One. 2012 Jul 2;7(7):e39869. doi: 10.1371/journal.pone.0039869 (PMC3388086; doi:10.1371/journal.pone.0039869)

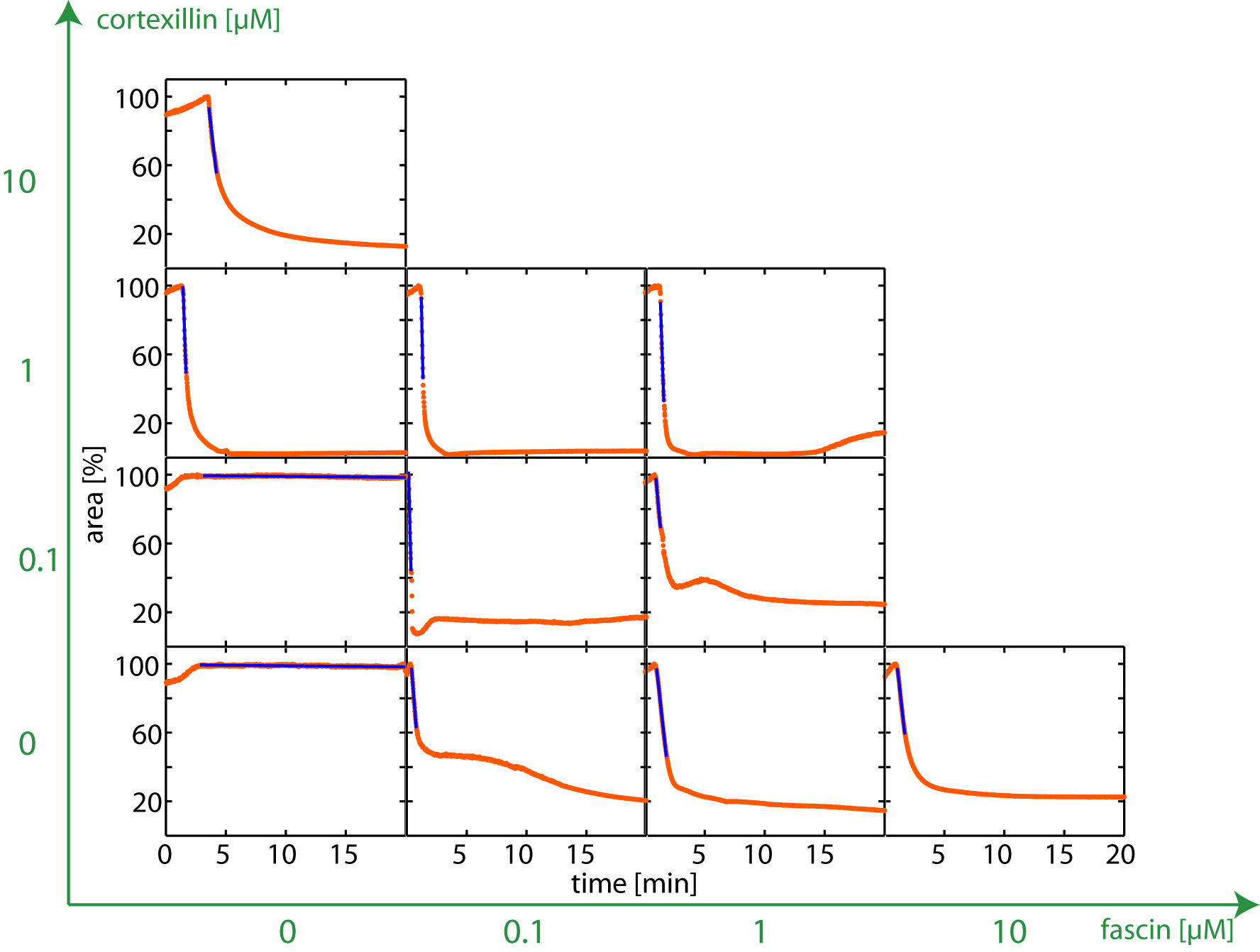

Supplement: Figure S1 — Dependence of the macroscopic contraction on the crosslinker concentration. The decrease in area over time (red dots) are shown for 10 M actin, 0.1 M myosin and crosslinking molecules at concentrations as indicated by the green axis. The area of the contracted region is normalized to the maximal area in the non-contracted state. Blue lines denote linear fits to initial contraction velocities. Initial increases of the area are due to spreading of the droplets. (TIF) [file pone.0039869.s001.tif]

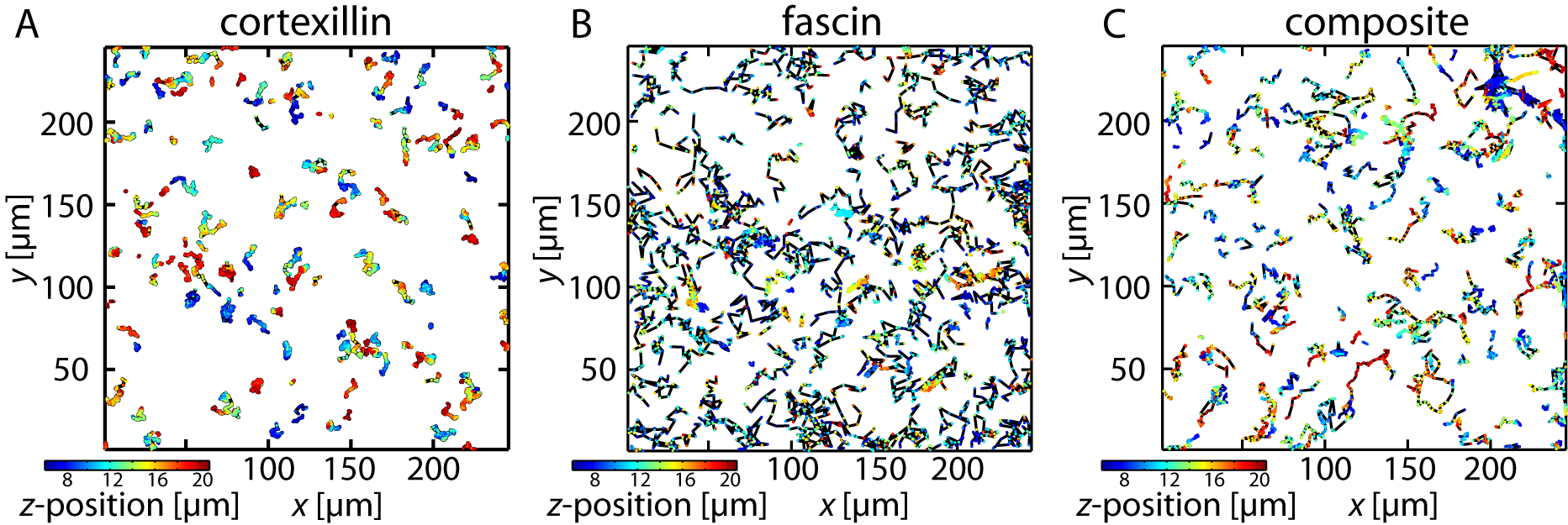

Supplement: Figure S2 — Cluster trajectories in active actin networks. Trajectories of clusters in active actin gels (3 M actin, 0.1 M myosin) are shown for networks crosslinked by 1 M cortexillin-I (A), 1 M fascin (B) and 0.5 M cortexillin-I and fascin, each (C). All trajectories exhibit stop and go motions. (TIF) [file pone.0039869.s002.tif]

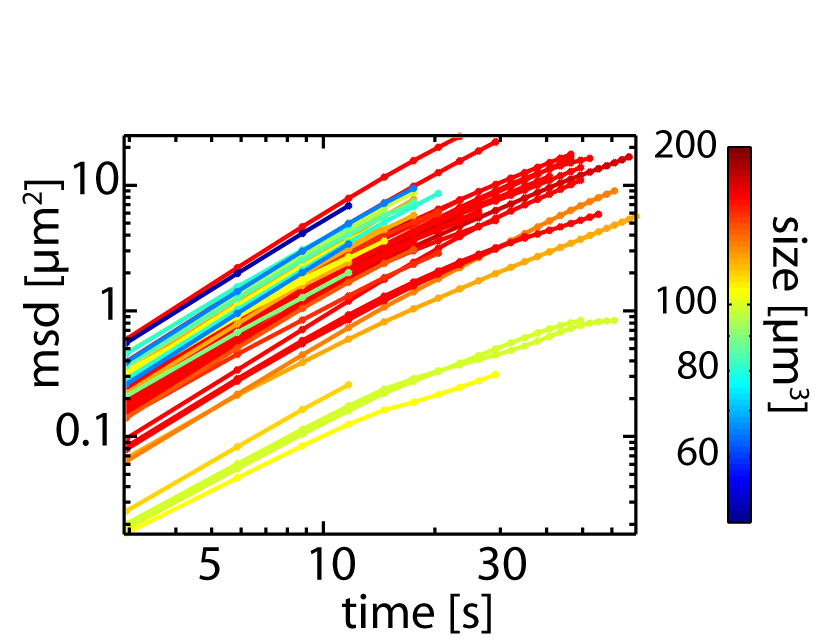

Supplement: Figure S3 — Dependence of the mean square displacement on the cluster volume. Colors from blue to red denote the cluster volume. All clusters show superdiffusive behavior which does not correlate with volume. (TIF) [file pone.0039869.s003.tif]

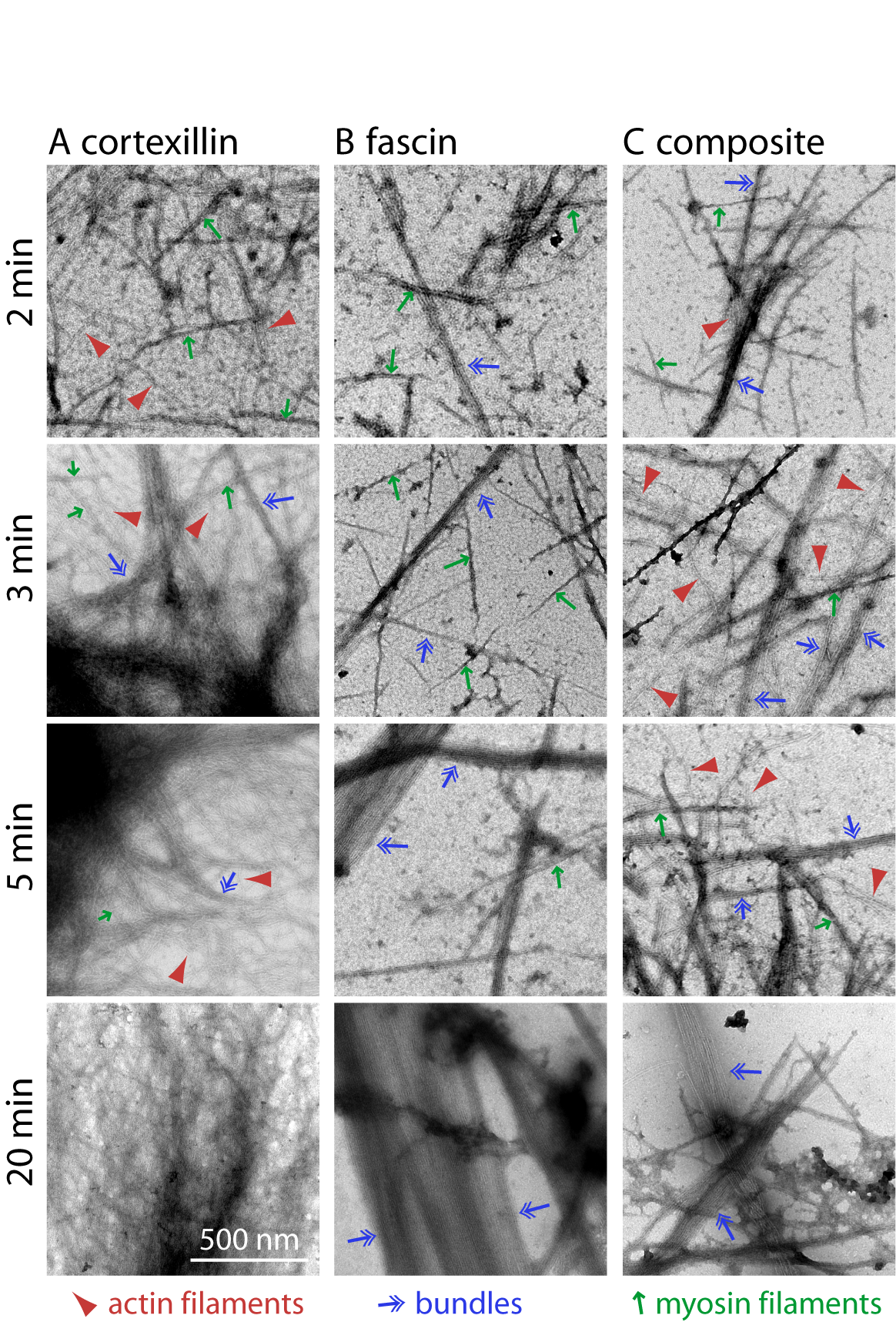

Supplement: Figure S4 — Time resolved electron micrographs of active actin networks. Electron micrographs are shown at 2 min, 3 min, 5 min and 20 min after initiation of polymerization for 3 M actin, 0.1 M myosin and 1 M cortexillin-I (A) or fascin (B) or 0.5 M fascin and cortexillin, each (C), respectively. Red arrowheads point to actin filaments, blue arrows indicate actin bundles and green arrows show myosin-II filaments. (TIF) [file pone.0039869.s004.tif]

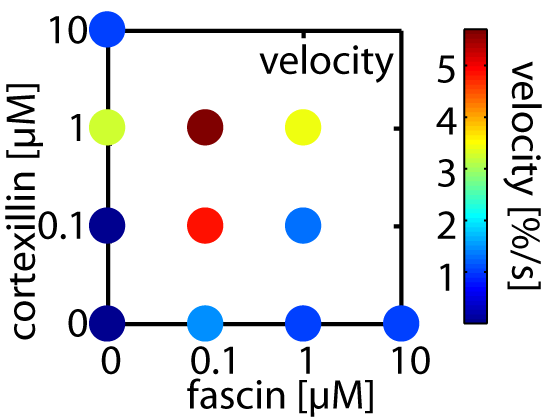

Supplement: Figure S5 — Phase diagram of the macroscopic contraction velocity. Initial velocities of contractions as shown in Fig. S1 are shown in dependence of crosslinker concentrations. (TIF) [file pone.0039869.s005.tif]

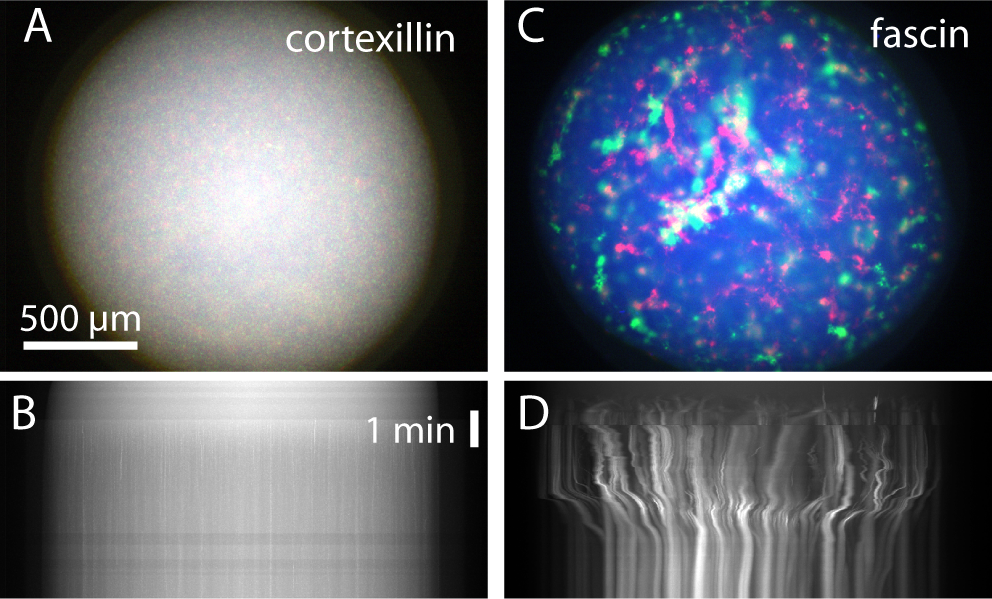

Supplement: Figure S6 — Contraction scenarios in composite active networks. A schematic overview of the contraction mechanisms in polar, apolar and composite active actin networks is shown. (TIF) [file pone.0039869.s006.tif]
